# GPCR Modulation in Breast Cancer

**DOI:** 10.3390/ijms19123840

**Published:** 2018-12-02

**Authors:** Rosamaria Lappano, Yves Jacquot, Marcello Maggiolini

**Affiliations:** 1 Department of Pharmacy, Health and Nutritional Sciences, University of Calabria, 87036 Rende, Italy; lappanorosamaria@yahoo.it; 2 Laboratoire des Biomolécules, CNRS-UM7203, Sorbonne University, Ecole Normale Supérieure, F-75252 Paris, France

**Keywords:** GPCRs, breast cancer, signal transduction

## Abstract

Breast cancer is the most prevalent cancer found in women living in developed countries. Endocrine therapy is the mainstay of treatment for hormone-responsive breast tumors (about 70% of all breast cancers) and implies the use of selective estrogen receptor modulators and aromatase inhibitors. In contrast, triple-negative breast cancer (TNBC), a highly heterogeneous disease that may account for up to 24% of all newly diagnosed cases, is hormone-independent and characterized by a poor prognosis. As drug resistance is common in all breast cancer subtypes despite the different treatment modalities, novel therapies targeting signaling transduction pathways involved in the processes of breast carcinogenesis, tumor promotion and metastasis have been subject to accurate consideration. G protein-coupled receptors (GPCRs) are the largest family of cell-surface receptors involved in the development and progression of many tumors including breast cancer. Here we discuss data regarding GPCR-mediated signaling, pharmacological properties and biological outputs toward breast cancer tumorigenesis and metastasis. Furthermore, we address several drugs that have shown an unexpected opportunity to interfere with GPCR-based breast tumorigenic signals.

## 1. Generalities

G protein-coupled receptors (GPCRs) are hepta-transmembranar proteins playing a crucial role in membrane-initiated signaling processes. They display a key role in a variety of physiological features including cardiac functions, immune responses, metabolism and neurotransmission. They may act alone or in collaboration with other membrane proteins such as, for example, steroid hormone receptors (SHR) [1] and tyrosine kinase receptors (TKR) [2]. As such, GPCRs participate in a plethora of autocrine and paracrine physiological effects.

Typically, the GPCR tertiary structure consists of seven highly hydrophobic transmembrane-spanning α-helices (namely I, II, III, IV, V, VI and VII) that are connected by three intracellular (ICL1, ICL2 and ICL3) and three extracellular (ECL1, ECL2 and ECL3) loops and that are spatially close to form an ~20 Å central cleft [3]. For instance, the gonadotrophin-releasing hormone receptor seems to be characterized by a physical contact between helix 2 and helix 7, through an asparagine (Asn-87) and an aspartate (Asp-318), respectively [4]. Whereas the N-terminal tail of GPCRs is extracellular, the C-terminal extremity is intracellular [5].

**Scheme 1:**
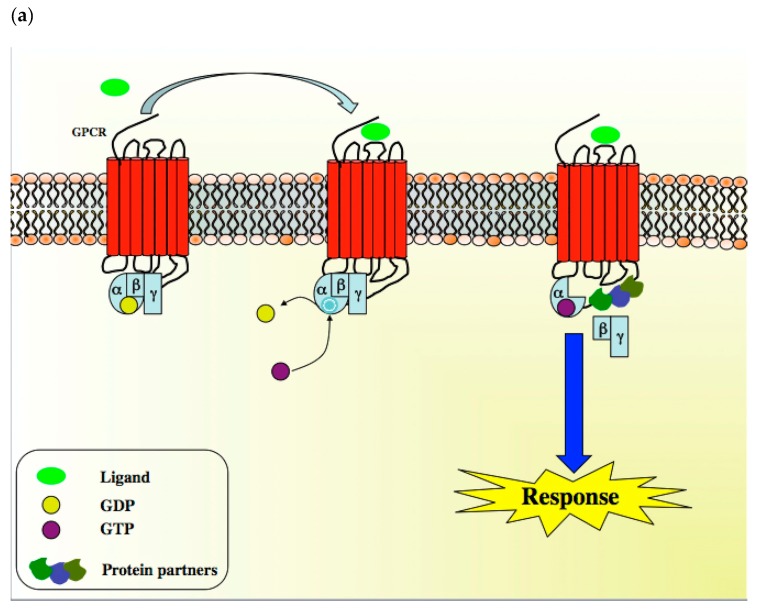

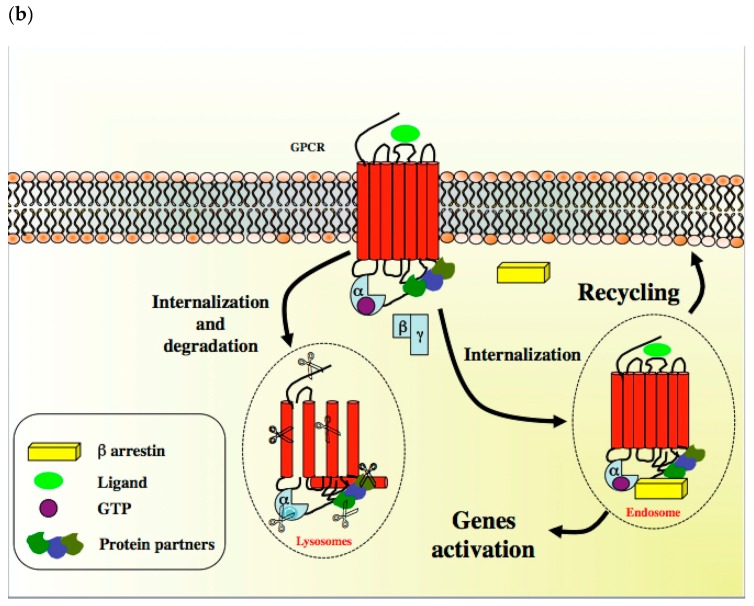
Activation and trafficking process of a G protein-coupled receptor (GPCR). (**A**) When a GPCR is activated, GTP takes the place of GDP at the GPCR-coupled G protein α subunit, allowing its release from the βγ subunit. Then, the complex of GPCR/agonist/α subunit/GTP activates a specific biological response, with the participation of specific partners. (**B**) As a regulatory (desensitization) process, β-arrestin is recruited, allowing GPCR translocation through endosomal pathways where it can be recycled at the membrane or translocated in the nucleus to activate specific genes. GPCR can also be internalized in lysosomes, allowing degradation.

A diverse array of GPCR ligands including inorganic ions, amino acids, peptides, proteins, steroids, lipids, nucleosides, nucleotides, biogenic amines and small molecules, as well as GPCR sensori stimuli such as light, tastes and odorants, transduce a wide range of extracellular signals into intracellular messages [6]. When a GPCR is activated, its conformation rapidly changes to allow it to couple to a Gα, Gβ and Gγ-containing heterotrimeric G-protein, which is anchored at the inner face of the plasma membrane through a myristyl, palmitoyl, farnesyl or geranyl lipidic chain (Scheme 1) [3]. Peptidic sequences also participate in G protein anchoring at the plasma membrane with the C-terminal part of the Gα_S_ subunit (sequence: Val–Asp–Thr–Glu–Asn–Ile–Arg–Arg–Val–Phe, residues 367 to 376) [7]. These conformational modifications lead to complex ligand-specific multi-states to induce dimerization and, in turn, discrete biological response. GPCRs can thus be considered as rheostats rather than classical off–on switches and their ligands can be considered as biased agonists since they can favor a specific conformation that is responsible for the activation of specific intracellular signaling transduction pathways (Scheme 1) [8,9]. In the GPCR basal state, the Gα subunit is bound to the guanine nucleotide GDP. In the presence of an agonist, GDP is replaced by GTP, allowing the dissociation of the Gα subunit from the βγ dimer (Scheme 1) [10,11]. Both subunit complexes prompt a network of intracellular effectors including second messenger generating systems, small GTPases and kinase cascades such as MAPK and PI3K/Akt, thus leading to changes in gene transcription and cellular events. Depending on the nature of the α subunit type (i.e., Gα_s_, Gα_i/o_, Gα_q/11_, Gα_12/13_), the coupling of GPCR to a G protein may affect diverse intracellular signaling pathways and determine distinct cell fates. To summarize, many GPCRs can stimulate multiple signaling systems and specific ligands can have different relative efficacies towards different transduction pathways. Ultimately, the integration of the functional activity of G protein-regulated networks modulates various cellular functions [12]. By phosphorylating specific serine and threonine residues in the C-terminus of GPCR, G protein kinases (GPK) abrogate GPCR-mediated action and allow the recruitment of β-arrestins, which are in charge of cytosolic internalization and degradation by lysosomes [3,13]. In addition to this regulatory process, GPCR can migrate from the cell surface to endosomes to activate specific genes [14] or can be recycled and sent back to the plasma membrane to display activity (Scheme 1) [15].

GPCRs constitute the largest superfamily of proteins in the human genome and one of the oldest family of membrane proteins. Their diversity seems to result from the recombination of well-defined amino acids sequences and from the rearrangement of domains that have occurred from a common ancestor, without altering signal functions. These modifications have led to a mosaicism with highly specific receptors, which are supported by a complex phylogenic network [16,17]. Based on their sequence homologies and poor sequence similarities, GPCRs are divided into five classes, i.e., rhodopsin (class A), secretin/adhesion (class B), metabotropic glutamate (class C), pheromone (class D), cAMP (class E) and frizzled/smoothened (class F) receptors [18].

In a pathological context, GPCRs are overexpressed and aberrantly activated, and as such are frequently implicated in many aspects of cancer, including tumor growth, invasion, migration, survival, angiogenesis and metastasis [19,20,21]. Given the appreciation of their role in cancer, the importance of GPCRs for anticancer drug discovery is undisputable, although very few members have been exploited in pursuit of anticancer therapies [22].

Breast cancer is a highly heterogeneous malignant disease with complex etiological and pathological characteristics. It is the most frequent cancer in women and a major public health problem, with approximately 1.7 million estimated new cases from population-based cancer registries in 2012 worldwide and more than 500,000 related deaths [23]. It has been predicted that the worldwide incidence of female breast tumors will reach 3.2 million new cases per year by 2050 [24], reflecting the sheer magnitude of breast cancer incidence, its effect on society and the urgent need for new treatments and preventive measures. Triple-negative breast cancers (TNBC), which constitute about 20% of all breast cancers, lack estrogen receptor (ER) and progesterone receptor (PR) expression and do not show gene amplification of the human epidermal growth factor receptor 2 (HER2) [25]. Moreover, they constitute a highly heterogeneous group of tumors that are characterized by various genetic alterations and a lack of validated biomarkers, but that share a distinctly aggressive profile with higher rates of relapse and shorter survival in the metastatic setting, compared to other subtypes of breast cancer [25]. Despite the recent development in subtyping TNBC and the promises of targeted therapies, therapeutic options are limited and cytotoxic chemotherapy remains the mainstay of treatment for these patients [26].

Here, we will discuss the current understanding of certain GPCRs that could be targeted to halt the growth of breast tumors, including TNBC, and that could constitute a new generation of diagnostic tools for breast cancer. In such a context, we will focus on GPCR-based drugs that have been designed, synthesized and evaluated for their efficacy in breast cancer.

## 2. GPCRs in Breast Cancer: Signaling, Biology and Modulators

### 2.1. GPER

**Figure 1:**
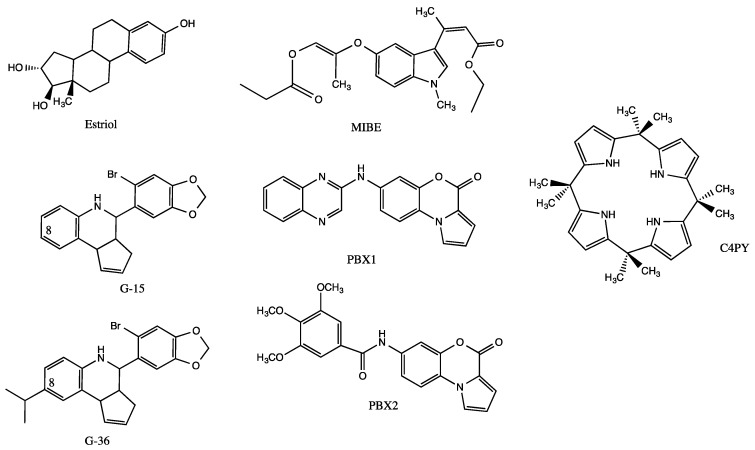
Structure of GPER antagonists.

Estrogens, which are required for normal breast tissue development but which play a well-established role in breast carcinogenesis, mainly act through the estrogen receptors named ERα and ERβ [27]. It is now well documented that estrogen signaling pathways may also occur through the G protein estrogen receptor GPER (originally called GPR30) [28]. This protein of 375 amino acids belongs to the rhodopsin-like (class A) receptor subfamily, as evidenced by the presence of an Asp–Arg–Tyr signature (residues 154 to 156, DRY motif) and an Asn–Pro–Xaa–Xaa–Tyr signature in the C-terminus of helix 7 (residues 320 to 324, NPxxY motif) [29,30,31]. Diverse experimental studies have demonstrated a role of GPER in the mediation of the stimulatory action prompted either by ER agonists including 17β-estradiol, phytoestrogens (e.g., genistein, coumestrol) and environmental estrogens (e.g., bisphenol A, dichlorodiphenyltrichloroethane) or ER antagonists such as tamoxifen, raloxifen and fulvestrant [32,33,34,35,36,37,38,39,40]. Synthetic selective and unselective GPER agonists (i.e., G-1, GPER-L1, GPER-L2, carbhydraz) and, as shown in Figure 1, antagonists (i.e., G-15, G-36, MIBE, C4PY, PBX1, PBX2) have been used to dissect the mechanisms governing GPER-mediated biological responses including those prompted in breast cancers [41,42,43,44,45,46,47,48]. Ligand-activated GPER produces rapid cellular signaling events including Ca^2+^ mobilization and kinase cascade activation via the transactivation of the epidermal growth factor receptor (EGFR) [48,49,50]. As a direct consequence, GPER regulates the expression of a plethora of genes involved in breast cancer cell growth and motility, which in turn are specifically inhibited by GPER antagonists [45,49,50,51].

Evidence for a role of GPER in breast tissue tumorigenesis and metastasis in vivo was provided using transgenic mouse models of mammary tumorigenesis (MMTV-PyMT, also called PyMT), where a GPER-null mutation (GPER KO/PyMT) was introduced. Tumors from GPER KO/PyMT mice were smaller, with decreased growth and metastatic potential when compared with GPER wild-type (PyMT phenotype) [52]. Accordingly, the tumors issued from GPER KO/PyMT mice were histologically of lower grade than those tumors issued from their wild-type (wt) counterparts, suggesting a less aggressive phenotype [52]. In connection with the above observations, it has been reported that the GPER antagonist G-36 inhibits agonist-induced cell proliferation in explants from normal and cancerous human breast tissues [53]. Analyses of GPER expression in primary breast tumor biopsies also indicate that it is directly linked to tumor size and metastases and, therefore, to pathological and clinical outcomes such as disease progression and poor survival [54]. In addition, GPER expression has been associated with tamoxifen resistance in breast tumors, which is consistent with numerous data indicating the stimulatory action of tamoxifen toward GPER [50,55,56,57].

Even if the GPER three-dimensional (3D) structure has not yet been experimentally resolved, structural information has emerged from computational approaches [58,59,60,61]. For instance, it has been demonstrated that a conserved proline was responsible for a kink in the seventh trans-membrane helix, allowing conformational changes and G-protein recognition [58]. Whereas the residues Met-1 to Phe-60 (in the N-terminus) correspond to the extracellular part of the protein, the residues Thr-330 to Lys-342, which are in the C-terminus (residues Thr-328 to Lys-375), point towards the inner face of the membrane. These GPER extracellular and intracellular regions are both composed of three loops, as classically observed in GPCRs. The C-terminal tail is in charge of the recruitment of G-protein and β-arrestin, whereas the Phe-208 to Val-225 second loop is essential for the recruitment of ligands [60]. It is of note that cysteins 130 and 207 form a disulfide bond participating in the binding site [60]. Independent studies have also shown that the GPER ligand-binding site is defined by the residues Val-116, Tyr-123, Met-133, Leu-137, Gln-138, Phe-206, Phe-208, Asp-210, Glu-275, Phe-278, Ile-279, Ile-308, Val-309, Phe-314 and Arg-394, where phenylalanines 206, 208 and 278 form a cluster [58,59,60,61]. Accordingly, estriol (E3, Figure 1) seems to interact with Thyr-123, Leu-137, Gln-138, Phe-206, Phe-208, Asp-210 and Glu-275 to exert antagonist effects [62]. In this regard, it should be stressed that conformational perturbations at the transmembrane helices II (residues Ala-110 to Ile-114), III (residues Met-141 to Ser-144) and VII (residues Ala-312 to Ser-315) seem to result from ligand binding, suggesting some protein flexibility [61]. 

Due to the heterogeneity of ligands and due to the absence of well-resolved crystal or NMR complexes, it is difficult to conclude about the structural requirements directing GPER agonism and antagonism. However, by being restricted to quinoleins, the molecule G-1 with an acetyl in position 8 displays agonist action, whereas the molecule G-36, which is characterized by a hindered hydrophobic isopropyl at the same position, is a GPER antagonist (Figure 1) [43,60]. Such an observation strongly suggests that a hydrogen bond-acceptor group in position 8 is required for GPER activation. As the antagonist G-15 is devoid of any substituent at carbon 8 of the quinolein moiety, it can be concluded that a hydrophobic substituent is not an absolute requirement for antagonist action. In fact, and according to modeling studies, substituents at position 8 could point towards cystein 207 to exert steric clash with Arg-394 [41]. The ligand G-15 seems to bind within a pocket of the GPER through an H-bond occurring between an oxygen atom of the methylenedioxyphenyl motif and Glu-54, as well as between the heterocyclic nitrogen of the quinolein moiety and Asn-310. Moreover, it could be involved in a tight salt bridge between the Asp-95 and Arg-155 of the conserved DRY key motif, which contributes to GPER folding and trafficking as well as to the recruitment of G-protein [30,61,63]. G-15 might also generate hydrogen bond with the asparagine 320 of the NPxxY (i.e., NPLIY) motif to stabilize the active state of the protein [63].

### 2.2. Lysophosphatidic Acid (LPA) and Sphingosine-1-Phosphate (S1P) Receptors

**Figure 2:**
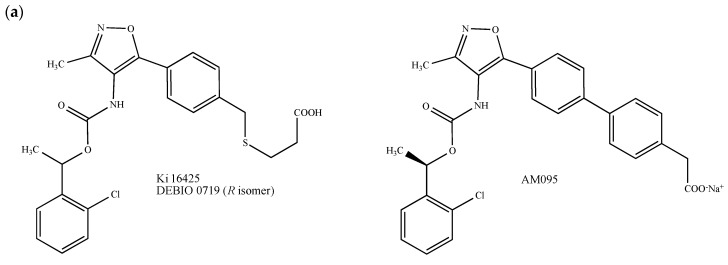

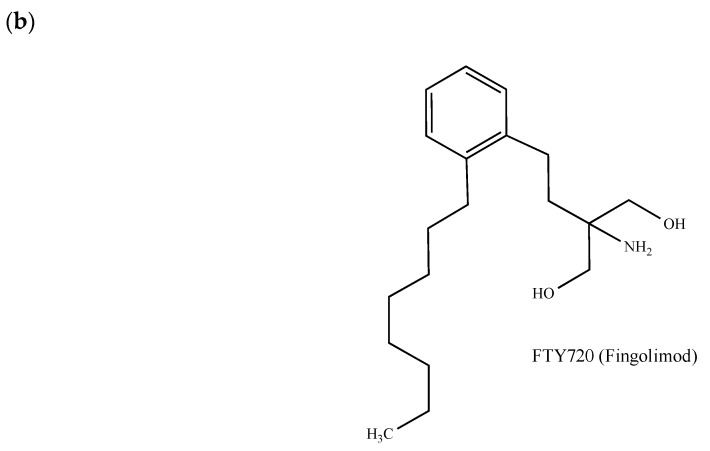
Structure of (**a**) lysophosphatidic acid (LPA) and (**b**) sphingosine-1-phosphate (S1P) antagonists.

Principally found in extracellular fluids, lysophosphatidic acid (LPA) and its endogenous derivatives exert pleiotropic effects including cell proliferation, migration, invasion, differentiation and adhesion, through at least six class A GPCRs, namely LPA1–6, which also play an important role in mediating malignant behaviors in breast cancer [64,65]. Due to the growing interest in LPA receptors and based on the fact that a well-conserved glutamine (Gln-125) present in the LPA1 binding-pocket governs the interaction with LPA and directs LPA/S1P receptors selectivity, small LPA receptors modulators mimicking LPA have been synthesized [66,67]. In particular, the LPA1/3 antagonist called Debio 0719 (Figure 2a) has been proposed as a promising molecule for breast cancer treatment. This compound, which corresponds to the *R*-isomer of the isoxazolic LPA1/3 competitive inhibitor Ki164425, exhibits higher antagonist effects for LPA1 and LPA3 (IC_50_ = 60 nM and 660 nM, respectively) than the parent molecule Ki16425 drawn in Figure 2a (IC_50_ = 130 nM and 2.3 μM, respectively) [68,69,70]. In vitro, Debio 0719 inhibits the LPA-dependent invasion of 4T1 mouse mammary cancer cells [69]. In BALB/c mice orthotopically inoculated with 4T1 cells in the mammary gland, the administration of Debio 0719 during the early phase of tumor growth reduces the number of spontaneously disseminated tumor cells to bone, lungs and liver [69,71]. Analogous results were observed using an experimental pulmonary metastatic model consisting of athymic NRC nu/nu mice inoculated with MDA-MB-231 human breast cancer cells [71]. Since Debio 0719 did not affect the growth of primary tumors and tumor-induced angiogenesis [69,71], it could be proposed, therefore, as a metastasis suppressor. Finally, the compound AM095, another Ki16425-derived isoxazole carboxylic acid derivative developed by the Amira Pharmaceuticals Company, showed interesting results on TNBC animal models (Figure 2a) [72].

Sphingosine-1-phosphate (S1P) is involved in tumor initiation, proliferation and metastasis as it behaves as an inflammation, neovascularization, cell growth and survival regulator [73]. This bioactive lipidic mediator mainly acts by interacting with and activating a family of five S1P-specific class A GPCRs (S1P1-5) [74]. The role for all S1P receptors in breast cancer was evidenced from studies demonstrating that their overexpression was linked with poor prognosis in breast tumor patients [75,76].

The simplicity of the structure of S1P offers a paradigm to chemists for pharmacomodulation. As previously highlighted for LPA1 [66], the polar part of S1P seems to be of prime importance for ligand-receptor interactions. Following computational studies, electrostatic contacts occurring between the two basic amino acids Arg-120 and Arg-292 and the phosphate group of S1P, as well as between the ammonium group and the residue Glu-121, have indeed been observed [77]. S1P analogues issued from rational modifications of the lipidic chain have also been synthesized (e.g., [78,79]). The functional S1P receptor antagonist FTY720 (Gilenya^®^, fingolimod, 2-amino-2-[2-(4-octylphenyl)ethyl]propan-1,3-diol) becomes active after its phosphorylation by the intracellular type 2 sphingosine kinase, an enzyme that is in charge of the conversion of sphingosine into S1P [80]. FTY720 (Figure 2b) not only reveals immunomodulatory properties (approved as such by the U.S. Food and Drug Administration (FDA) for the treatment of multiple sclerosis), but has also proven efficacy in multiple in vitro and in vivo cancer models including breast tumors [81,82,83,84]. However, due to its immune suppressive effects, its future use in oncology seems to be strongly compromised. The potential anticancer mechanism of FTY720 may occur through the inhibition of the proto-oncogene enzyme sphingosine kinase 1 [85] and other targets including the protein phosphatase 2A, cell cycle regulators, cell transporters, autotaxin and the mitochondrial permeability transition pore [85]. 

Lastly, it should be noted that some information has emerged from structural studies. It has been demonstrated that LPA and SP1 receptors share strong structural similarities but limited local differences [86]. The N-termini of LPA1 and SP1 receptors differ by the presence of a stabilizing disulfide bond occurring between the N-terminal capping helix and the second extracellular loop. Likewise, the short fragment located between the transmembrane helix 1 and the N-terminal capping helix adopts a helical conformation in SP1 but not in LPA1, where it is unstructured [86]. Such structural differences could partially explain not only LPA1/S1P selectivity, but also why the protein LPA1 accepts more structurally divergent ligands than the SP1 receptor.

### 2.3. Prostaglandin E2 Receptors

An aberrant overexpression of the cyclooxygenase (COX)-2, which occurs in 40–50% of invasive breast cancer patients, is associated with a worse prognosis [87]. Likewise, the major COX-2 product found in the tumor milieu is the prostaglandin E2 (PGE2). By acting on a family of four GPCRs (i.e., EP1–4) and more specifically on EP1 and EP4, PGE2 promotes multiple cellular events including the inactivation of host antitumor immune cells, the enhancement of cancer cell migration and invasiveness, tumor-associated angiogenesis and lymphangiogenesis. Thus, it has been postulated that EP antagonists could be of interest for the control of the progression of mammary tumors [87]. Accordingly, non-steroidal anti-inflammatory drugs (NSAIDs) and selective COX2 inhibitors have been shown to prevent the growth of experimental breast tumors (see [87] and the references therein). 

**Figure 3:**
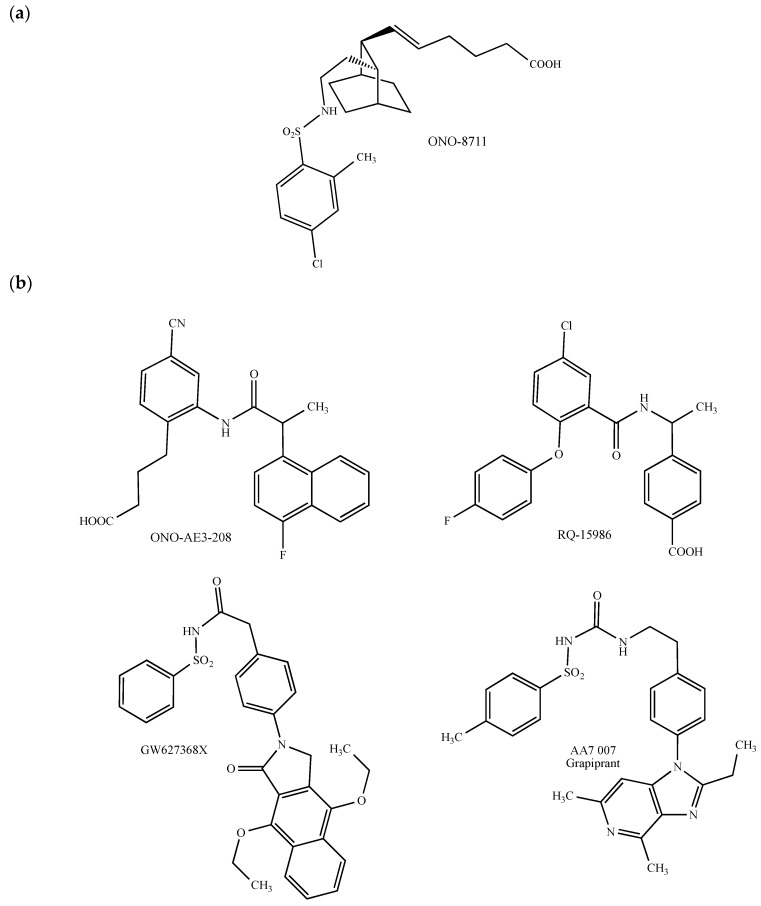
Structure of (**a**) EP1 and (**b**) EP4 antagonists.

The EP1 inhibitor ONO-8711 (Figure 3a) administrated at the dose of 800 ppm in female Sprague–Dawley rats exerts apoptotic effects in PhIP (2-amino-1-methyl-6-phenylimidazo [4,5-*b*] pyridine)-induced mammary gland tumors [88].

EP4, which is widely expressed in primary invasive ductal breast carcinomas, functions through cAMP and PKA pathways and activates PI3K. Accordingly, the blockade of EP4 by diverse small molecules including AH23848, ONO-AE3-208, GW627368X and RQ-15986 (Figure 3b), as well as the triterpenoid saponine Frondoside A, inhibits the proliferation and migration of breast tumor cells, prevents phenotype changes of breast cancer stem cells and reduces breast tumor-initiating capacity, growth and metastasis [89,90,91,92,93,94,95,96]. Taking into account that EP4 antagonists at therapeutic doses are well-tolerated, a phase II trial is currently evaluating the potential of the GW627368X-derived EP4 antagonist AAT007 (also known as RQ-07 or grapiprant, Galliprant^®^, Figure 3b) with respect to circulating tumor cells and the improvement of outcome in advanced prostate, breast and non-small cell lung cancer alone or in combination with gemcitabine. As EP4 is now identified as a promising new therapeutic target for breast cancer, more potent antagonists such as AAT-008 [97], which show improved pharmacological profiles and bioavailability, have recently been identified.

### 2.4. Protease-Activated Receptors

Coagulant factors such as thrombin and tissue factor (TF) are generated in the tumor microenvironment independently of blood coagulation and induce cell signaling responses through the activation of protease-activated receptors (PARs), which play important roles in neural tube closure, hemostasis, inflammation and the vascular system [98]. PARs also mediate cancer invasion and metastasis by promoting tumor cells migration and angiogenesis and by facilitating cancer cell interactions with host vascular cells including platelets, fibroblasts and blood vessels lining endothelial cells [98]. More precisely, PAR1 and PAR2 have been identified as mediators of breast cancer cell invasion and migration [99,100].

**Figure 4:**
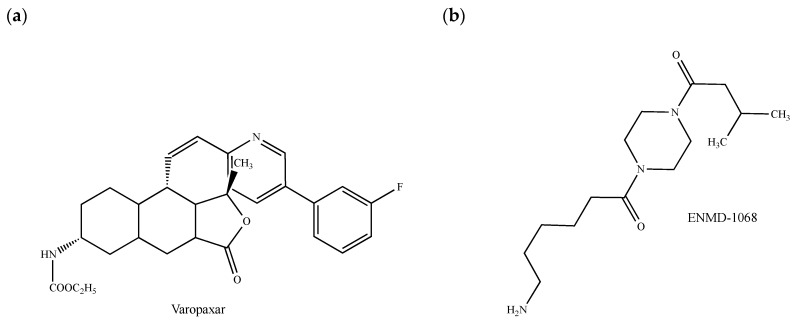
Structure of (**a**) PAR1 and (**b**) PAR4 antagonists.

The commercially available PAR1 antagonist SCH 530348 (vorapaxar, Zontivity^®^, Figure 4a) has been approved by the FDA to reduce thrombotic cardiovascular events in patients with myocardial infarction and peripheral artery disease history but without previous stroke or transient ischemic attack [101]. This drug, which reduces thrombin-induced ovarian cancer cell proliferation [102] and which interferes with the growth and migration of glioma tumor-initiating progenitor cells [103], has not yet been evaluated in breast cancers despite potential advantages in this pathological context. The PAR1 inhibitor PZ-128 (palmitoyl–Lys–Lys–Ser–Arg–Ala–Leu–Phe–NH_2_ or P1pal7) belongs to the new family of cell-penetrating membrane-tethered lipopeptides (namely pepducins), which are inspired from the intracellular loops located at the GPCR/G-protein interface [104]. PZ-128 is in a phase II clinical trial for its preventive effects against ischemic and thrombotic complications in patients undergoing cardiac catheterization. Strikingly, it also reduces PAR1-driven tumor growth, angiogenesis and metastatic lesions in breast cancer xenografts [105,106]. Although the question remains whether PZ-128 and other PAR1 inhibitors could be used for the treatment of metastatic breast cancer, pepducins might block the receptor-mediated activation of one specific signaling pathway without affecting others by targeting diverse intracellular loops of PAR1 [107]. In this regard, it should be stressed that the peptidic sequence Pro-Phe-Ile-Ser-Glu-Asp, which has been designed from the *Vibrio cholerae* hemagglutinine protease-mediated cleavage of the mouse PAR1 protein, displays apoptotic effects in MCF-7 breast carcinoma cells by interfering with PAR1 itself, but at a site different from the thrombine-mediated activation site [108].

Concomitant with an increase of the amount of vascular endothelial growth factor (VEGF) and in correlation with enhanced metastatic potential, PAR2 plays also a major role in breast cancer cell proliferation, migration and invasion [100,109]. From a clinical point of view, the elevated expression of PAR2 detected in breast tumor biopsy and metastatic tissues could be linked to an increased malignancy grade and, subsequently, to an overall decrease of the survival rate, as also observed with PAR1 [110]. Some of the PAR2 antagonists also present antitumor properties [111,112]. This is the case of ENMD-1068 (Figure 4b), which is the only commercially available PAR2 antagonist. ENMD-1068 decreases in MCF-7 breast cancer cells the concentrations of granulocyte colony-stimulating factor (GCSF), a highly expressed cytokine correlated with poor survival [113,114]. Recently, structure–activity relationship studies have allowed the identification of promising selective PAR2 inhibitors including derivatives of teleocidin with high efficacy in inhibiting breast tumor cell migration [115]. Whether PAR2 antagonists could be considered as PAR1 antagonists and vice versa is a matter of interest given that PAR2 interacts with PAR1 to form a functional unit implied in breast tumor development [116].

Lastly, the pleckstrin homology (PH)-domain-binding motifs in the C-tails of PAR1 and PAR2, which have been recently recognized as important for PAR-driven breast tumor growth, could serve as novel platforms for future drug therapy design [117].

### 2.5. Chemokine Receptors

One of the main chemokine receptors involved in breast growth and metastasis is the type 4 C-X-C chemokine receptor (CXCR4, where C corresponds to cysteine), which accepts for the endogenous ligand the stromal-derived-factor-1 SDF-1 (also called CXCL12) [118,119,120,121].

**Figure 5:**
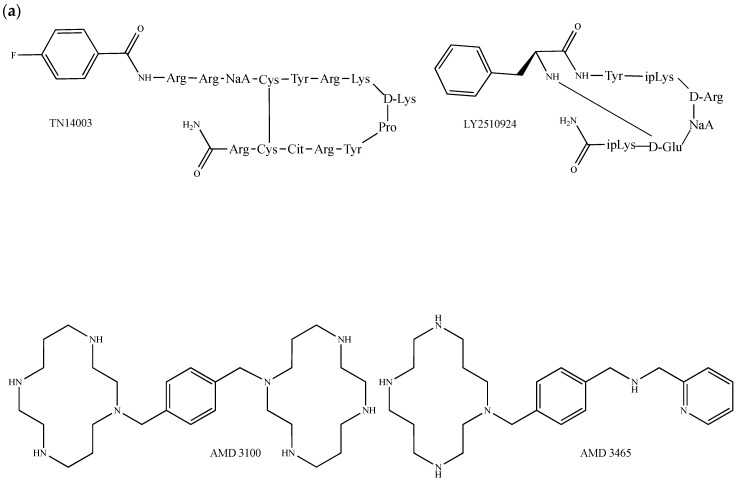

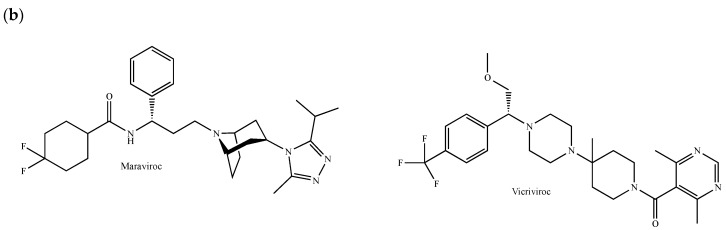
Structure of (**a**) CXCR4 and (**b**) CXCR5 peptidic and heterocyclic inhibitors. NaA and ipLys correspond to l-3-(2-naphtyl)alanine and *N*-isopropyl-l-lysine, respectively.

A variety of peptides and small molecules targeting CXCR4 attenuate the growth of breast cancer both in vivo and in vitro. The non-natural 14-mer cyclic peptide TN14003 (Figure 5a), which contains a d-lysine and a proline to induce a turn conformation stabilized by a cysteine-mediated disulfide bond, was bioinspired from the truncated polyphemusin peptide analogue T140, a CXCR4 specific inverse agonist with anti-HIV properties [122,123]. Remarkably, this compound has the capability to prevent VEGF-mediated tumor angiogenesis induced by the CXCR4/CXCL12 axis in breast tumor xenografts [124] and exerts anti-metastatic activity in breast cancer in cultured cells and animal models [125,126,127]. The other anti-HIV SDF-1 competitor, Nef-M1, an apoptotic peptide that encompasses the residues 50 to 60 of the HIV-1 Nef protein (sequence: Thr–Asn–Ala–Ala–Cys–Ala–Trp–Leu–Glu–Ala–Gln), not only inhibits the growth of primary breast tumors and related metastasis [128,129], but also prevents breast tumor angiogenesis and epithelial-to-mesenchymal transition [130]. The CXCR4 antagonist GST–NT21MP, a 21-mer synthetic peptide derived from the N-terminal extremity of the viral macrophage inflammatory protein II, also called vMIP-II (NT21MP sequence: Leu–Gly–Ala–Ser–Trp–His–Arg–Pro–Asp–Lys–Cys–Cys–Leu–Gly–tyr–Gln–Lys–Arg–Pro–Leu–Pro, residues 1–21), abrogates SDF-1-induced cell growth, breast cancer cells adhesion and migration, and delays pulmonary metastasis in vivo [131,132]. The SDF-1 peptide analogue CTCE-9908, which consists of a dimer of the first eight amino acids of SDF-1 and in which each Lys–Gly–Val–Ser–Leu–Ser–Tyr–Arg monomer is linked by a C-terminal amidated lysine, behaves as an SDF-1 competitive inhibitor. CTCE-9908 reduces the growth of primary breast tumors and metastasis and markedly enhances the efficacy of other commonly used anticancer therapies such as antiangiogenic (anti-VEGF) antibody and cytotoxic agents (e.g., docetaxel) in mice breast cancer models [133,134,135]. ALX40-4C (N-α-acetyl-nona-d-arginine amide acetate), which selectively blocks the interaction of SDF-1 with CXCR4, inhibits breast carcinoma cell invasion without decreasing cell viability [136], suggesting a distinct contribution of CXCR4 to the invasion but not to the survival of breast cancer cells. The small cyclic peptide LY2510924 (Figure 5a), which is currently in phase I and II clinical studies for the treatment of advanced refractory solid tumors, inhibits breast tumor metastasis by blocking the migration/homing process to the lung and by inhibiting cell proliferation after tumor cell homing [137]. Among the small CXCR4 antagonists, the cyclam AMD3465 (Figure 5a) inhibits breast cancer growth and metastases by acting on both tumor and immune cells [138]. Furthermore, it reduces breast cancer cell invasiveness and the formation of breast tumors and metastases as well as the infiltration of myeloid CD11b positive cells at metastatic sites and spleen tissue [138]. During the last decade, great interest has particularly been paid to the AMD3465-derived molecule AMD3100 (plerixafor, Mozobil^®^, Figure 5a), a bicyclam that selectively and reversibly binds within the CXCR4 to disrupt tumor-stroma interactions and that mobilizes hematopoietic stem and progenitor cells to the peripheral blood compartment [139]. Following molecular modeling studies performed with the human protein CXCR4, it was found that the interaction of AMD3100 and AMD3465 with CXCR4 probably results from electrostatic interactions involving the amino acids Asp-171 (transmembrane helix 4), Asp-262 (transmembrane helix 6) and Glu-288 (transmembrane helix 7). Furthermore, one cyclam could interact with Asp-171, whereas the other is sandwiched between the Asp-262 and Glu-288 carboxylic acid functions [140]. The mode of binding of AMD3100 and AMD3465 presents similarities with LY2510924, which requires the residues His-113, Asp-187, Arg-188, Phe-189, Tyr-190, Gln-200 and Glu-288 [137]. Importantly, the FDA has approved AMD3100 for patients with multiple myeloma and non-Hodgkin lymphoma. In the context of breast cancer, AMD3100 (i) inhibits the SDF-1-induced activation of diverse oncogenic signals such as the JAK2/STAT3 signaling pathway [141], (ii) increases cellular sensitivity to carboplatin [142], (iii) cooperates with a pure antiestrogen to decrease breast cancer cell proliferation and migration induced by mesenchymal stem cells [143] and (iv) attenuates hypoxia-dependent metastatic potential [144]. In addition, it limits lung metastases from orthotopically transplanted breast cancer cells [145], abolishes wound-promoted tumor growth and decreases collagen deposition and neo-angiogenesis in mouse models of breast cancer [146]. More recently, AMD3100 has been recommended as a potent radiosensitizer as it augments TNBC cell radiosensitivity and irradiation-induced tumor growth delay [147]. Lastly, the CXCR4 protein epitope POL5551 has been shown to disrupt metastasis and enhance the chemotherapeutic effects in TNBC [148]. In the same context, the monoclonal antibody Ulocuplumab (BMS-936564 or MDX1338) has shown promising anti-CXCR4 action [149].

The chemokine ligand 5 (CCL5)/chemokine receptor 5 (CCR5) system also plays a role in promoting breast cancer onset and progression [150]. CCL5 is, indeed, implicated in bidirectional communication patterns between cancerous and normal breast cells as it can be secreted either by tumor or mesenchymal cells that are recruited to the tumor [150]. Moreover, CCR5, which is highly expressed in specific subtypes of breast tumors, has been shown to control breast cancer cell invasiveness and associated metastasis [151].

Maraviroc (Selzentry^®^, Figure 5b) is the only CCR5 inverse agonist currently approved by the FDA, the European Commission, Health Canada and several other organizations for the treatment of HIV-1-infected patients carrying a CCR5 tropism [152]. Based on the human CCR5 crystal structure, it has been shown that maraviroc occupies a pocket delineated by helices I, II, III, V and VI, where the residues Glu-283 and Tyr-251 interact with the nitrogen atom of the tropane bicyclic motif and the carboxamide nitrogen, respectively [153]. The threonines 295 and 259 also participate in interactions with one of the fluorine atoms and the cyclohexane [153]. The phenyl group fits within an aromatic sub-pocket defined by Phe-109, Phe-112, Tyr-208, Trp-248 and Tyr-251 [153]. Vicriviroc (SCH 417690) is another antiretroviral agent blocking CCR5 (Figure 5b). It shares good oral bioavailability, long half-life, minimal toxicity and excellent antiviral properties in patients infected with CCR5-tropic HIV-1 [154]. Remarkably, the inhibition of CCR5 by maraviroc and vicriviroc prevents breast cancer cell invasiveness, enhances breast cancer stem killing mediated by chemotherapeutic agents targeting DNA and reduces tumor growth, angiogenesis and metastatic colonization in vivo [151,155,156].

**Figure 6:**
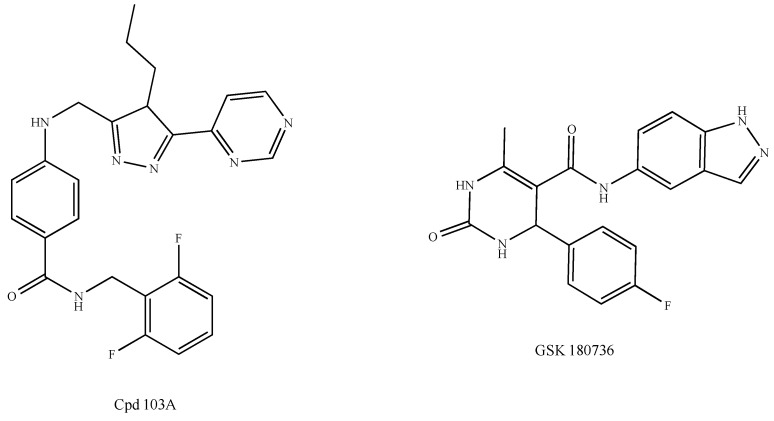
Structure of the GRK2 inhibitors 103A and GSK180736.

The protein CXCR4 is under the control of the G protein-coupled receptor kinases GRK2 and GRK3. They correspond to negative GPCR regulators that are in charge of the phosphorylation of specific serine and threonine residues prior to the recruitment of β-arrestins to allow receptor internalization (desensitization) and degradation (Scheme 1b) [118]. New approaches devoted to the development of antitumor drugs targeting GRKs have recently been under consideration. As emerging oncomodulators and focusing on ligand-bound GPCRs, only GRKs may prevent the hyperactivation of GPCRs and, therefore, cell proliferation and migration [157]. However, GRK2 induces the activation of the histone deacetylase HDAC6 and, therefore, the growth of luminal and basal breast tumors through Pin1, which is in charge of the *cis*-*trans* isomerization of the ERα Ser-218 [158,159]. Thus, the inhibition of GRK2 could open a new perspective for the treatment of breast cancer in combination with other chemotherapeutic molecules simultaneously targeting HDAC6 and Pin1 pathways. In this context, GRK2 inhibitors such as the compound 103A have been developed by Takeda Pharmaceuticals (Figure 6) [160]. Likewise, the paroxetine derivative GSK180736A has shown promising results (Figure 6) [161]. GRK3 could also constitute a promising target since it is an important actor in TNBC metastasis [162].

Finally, it should be noted that an increase of the CXCR4:GRK3 ratio has been shown to reflect a low metastatic potential in TNBC as it correlates with high amount of CXCR4 and low amount of GRK3 [162]. Hence, CXCR4 as well as GRK3 could be used as biomarkers, not only for the diagnosis of breast tumors but also to define phenotypes and evaluate metastatic risks.

### 2.6. Angiotensin II Type 1 Receptor

**Figure 7:**
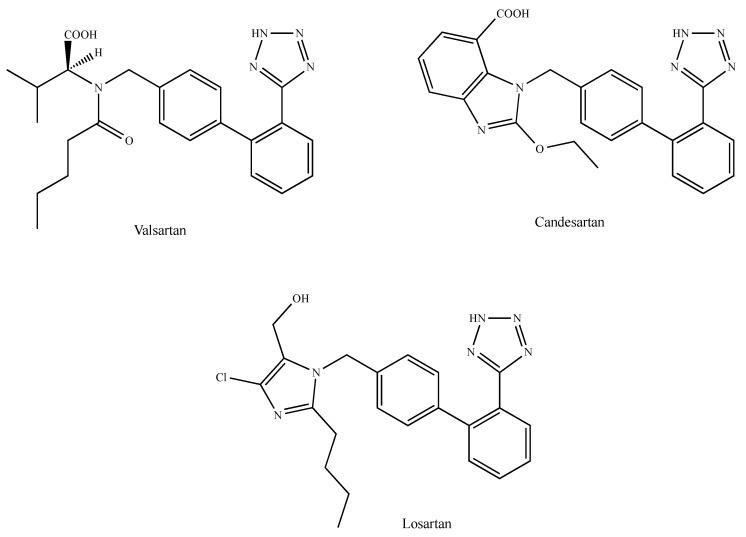
Structure of the AT1R antagonists valsartan, candesartan and losartan.

The angiotensin II (AngII) octapeptide is a major actor of the renin-angiotensin system (RAS). Apart from its physiological function, it plays a role in diverse tumors including breast cancer [163]. AngII is produced by the cleavage of the inactive decapeptide precursor angiotensin I (AngI) by a zinc metalloprotease found in the blood circulation or bound to the cell membrane and known as AngI-converting enzyme (ACE) [164]. AngII-mediated biological effects occur through binding to the class A G protein-coupled AngII type 1 and type 2 receptors (AT1R and AT2R, respectively), which have different tissue distribution and which activate various signaling pathways [164]. Most AngII-mediated effects have been attributed to AT1R stimulation, whereas AT2R mainly acts as a counter-regulatory receptor [164]. AngII/AT1R signaling triggers a variety of intracellular effectors, leading to the regulation of tumor progression-related processes such as cell proliferation, migration, angiogenesis, inflammation and tissue remodeling [165]. AT1R is overexpressed in malignant breast tissue, where it promotes EMT and invasion in vitro, as well as tumor growth and angiogenesis in vivo [166]. Recent interest has focused on the potential anticancer action of ACE inhibitors, which have been successfully used as potent antihypertensive drugs over the last 30 years [167]. By reducing the production of AngII, ACE inhibitors indirectly block AT1R- and AT2R-mediated signaling pathways. However, direct AT1R blockers (ARBs) produce better anticancer clinical results [168]. Molecular modeling studies have revealed that selective AT1R blockers interact at diverse AT1R sites [169]. For instance, the carboxylic acid group and tetrazole ring of valsartan (Figure 7) possibly interact with the Lys-199 of the transmembrane domain TM5, the Ser-109 of the TM3 and the Asn-295 (TM7) of the AT1R, respectively. The carboxylic group, the tetrazole ring and the ethoxy group oxygen of candesartan (Figure 7) seem to interact with the Lys-199 of TM5, the Ser-109 of TM3, the Asn-295 of TM7 and the Gln-257 of TM6 [169]. In the same context, the tetrazole ring and the hydroxymethyl groups of losartan (Figure 7) possibly interact with the Asn-295 of TM7 and the Ser-109 of TM3, respectively [169]. In accordance with the above observations, candesartan is the antagonist that shares the highest binding affinity for AT1R, while losartan shows the weakest affinity as it has the lower number of anchoring points [170]. As it concerns the role displayed by AT1R blockers in the context of breast cancer, it should be noticed that both candesartan and losartan inhibit the AngII-induced expression of VEGF-A, whose expression, along with that of AT1R, was found to correlate with an increase of the microvascular density in breast cancer patients [171]. Moreover, losartan blocks breast tumor growth, invasion, angiogenesis and EMT prompted by AT1R overexpression [166,172], inhibits mammary tumor development and progression to invasive carcinoma [173] and potentiates the doxorubicin and paclitaxel-induced inhibitory effects on breast cancer growth and metastasis [174,175,176]. The increase of AT1R levels in tamoxifen-resistant breast tumor cells, as compared to sensitive counterparts and tamoxifen sensitivity, were restored by the blockade of AT1R by losartan (Cozaar^®^) [177]. Lastly, it is noteworthy that antineoplastic effects are also exerted by further AT1R blockers such as telmisartan (Micardis^®^) and irbesartan (Avapro^®^) [178,179]. Taken together, these results support the concept of a role for AT1R antagonists in suppressing breast cancer development and progression.

### 2.7. Gonadotropin-Releasing Hormone Receptors

By binding to the specific well-conserved class A seven-transmembrane receptor GnRHR, the gonadotropin-releasing hormone GnRH (or luteinizing hormone-releasing hormone LHRH), which is produced in the hypothalamus in a pulsatile manner, stimulates the secretion of gonadotropin when present in the pituitary, thus regulating steroidogenesis and gametogenesis [180]. Briefly, GnRH is a neuroendocrine decapeptide issued from the proteolysis of a preprohormone of 89 amino acids. Its sequence (pyroGlu–His–Trp–Ser–Tyr–Gly–Leu–Arg–Pro–Gly–CONH_2_) contains a non-proteinogenic pyroglutamic acid (i.e., 5-oxoproline) in the N-terminus and an amidation in the C-terminus.

GnRH analogues induce the downregulation of GnRHR and, therefore, desensitization, whereas competitive antagonists block the secretion of gonadotropin with immediate effects and the absence of hormonal flare (Scheme 1b). Thus, both strategies, which ultimately reduce the amount of circulating gonadotrophins and gonadal steroids, could be used for the treatment of hormone-dependent neoplasms such as those of the prostate, ovary, endometrium and breast [181], in accordance with GnRHR overexpression in extrapituitary steroid-dependent neoplastic tissues [182]. In this regard, it should be stressed that the activation of GnRHRs reduces the cell proliferation and metastasis of cancer cells, indicating a possible direct antitumor activity of GnRH analogues [183]. The suppression of ovarian functions with synthetic GnRH analogues (i.e., agonists) and antagonists has been extensively studied in premenopausal women with early-stage and advanced breast cancers [184].

**Figure 8:**
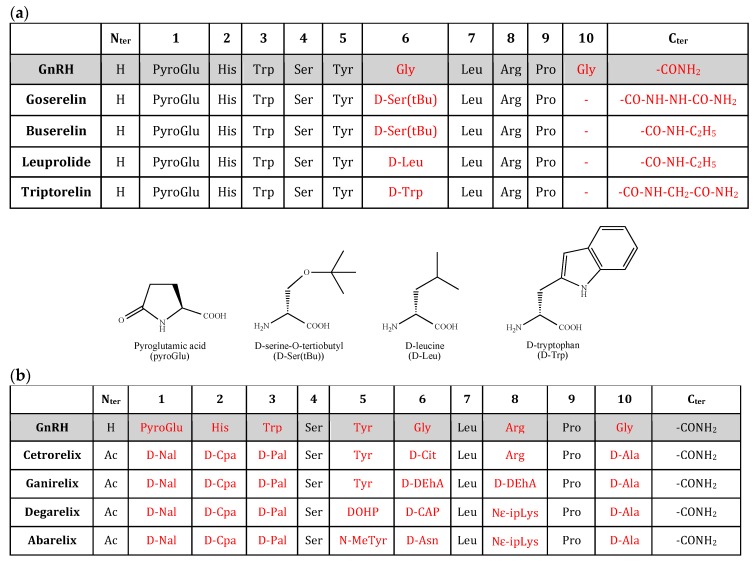

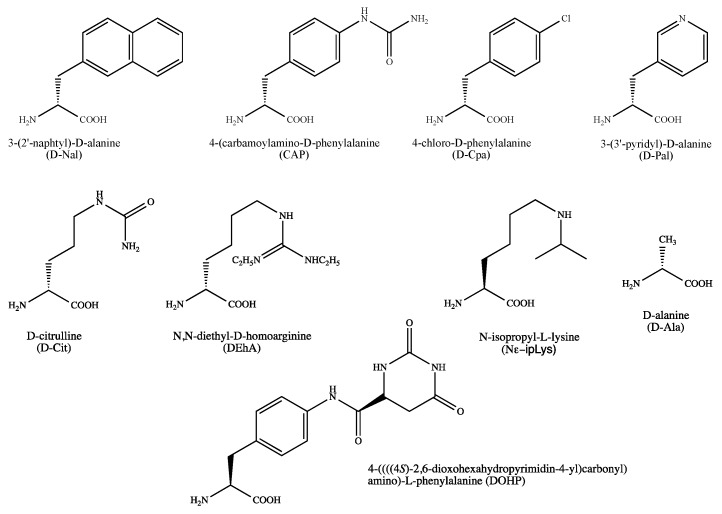
Structure of (**a**) GnRHR agonists (goserelin, buserelin, leuprolide and triptorelin) and (**b**) GnRHR antagonists (cetrorelix, ganirelix, degarelix, and abarelix). The residues that are subject to pharmacomodulation are indicated in red. The structures and symbols of the non-proteinogenic amino acids of these molecules are specified.

GnRH peptidic analogues such as goserelin (Zoladex^®^), buserelin, leuprolide (leuprolein, Eligard^®^) and triptorelin (Triptodur^®^) have proven to be as effective as surgical oophorectomy in premenopausal advanced breast cancer [185,186,187,188]. As such, they offer similar outcomes compared with tamoxifen, although the endocrine combination appears to be more effective than GnRHR agonists alone [189]. In the case of GnRH analogues, the principal chemical modifications concern the glycine in position 6 as well as the C-terminal extremity. As the GnRH secondary structure is associated with a turn centered around the Gly-6, strategies consisting in introducing conformational constraints to stabilize this turn have been successfuly exploited [190,191]. This glycine has been substituted by d-amino acids such as, for example, a d-Ser(tBu) (e.g., goserelin and buserelin), a d-Leu (e.g., leuprolide) or a d-Trp (e.g., triptorelin), as shown in Figure 8b. Amidation in the C-terminus has also been shown to improve the affinity of the ligands for the receptor [190,191]. Thus, chemical modifications introduced in the GnRH peptidic sequence allow both conformational constraints and affinity improvement, and contribute to improvements of the stability against proteolysis [191]. At present, goserelin is the only GnRH analogue approved by the FDA for the treatment of ER-positive premenopausal metastatic breast cancer [192]. Goserelin, which is a biodegradable sustained-release 3.6 mg depot administered by subcutaneous injection every 28 days, is well-tolerated and associated with less acute toxicity than cytotoxic chemotherapy. Numerous clinical trials in premenopausal women with hormone-sensitive early breast cancer have demonstrated that goserelin either alone or in combination with tamoxifen is at least as effective as cyclophosphamide-, methotrexate- or 5-fluorouracil-based chemotherapy. Likewise, the combination of triptorelin with the anti-aromatase formestane has shown increased antitumor effects [193].

Potent peptidic GnRHR antagonists such as cetrorelix (Cetrotide^®^), ganirelix (Orgalutran^®^), degarelix (Firmagon^®^) and abarelix (Plenaxis^®^) have been synthesized and clinically tested [194]. To enhance their stability and to improve selectivity in vivo, these compounds share non-proteinogenic amino acids and lack the N-terminal pyroglutamic acid, which is a hallmark of GnRH analogues. GnRH antagonists act by competing with native GnRH in the receptor binding sites, thus causing an immediate suppression of the release of gonadotropins and sex steroids. As such, they have been evaluated in controlled ovarian hyperstimulation protocols for the prevention of premature luteinizing hormone surge [195] and are successfully used to treat advanced prostate cancer [196]. GnRH antagonists are well-tolerated not only in male but also in female cancers (i.e., endometrial, ovarian and breast cancers) [197]. GnRHR antagonists can induce breast cancer cell apoptosis via the activation of stress-induced mitogen-activated protein kinases and pro-apoptotic proteins [197], as exemplified with cetrorelix, which inhibits the growth of TNBC tumors xenografted in nude mice [198]. In combination with aromatase inhibitors, GnRH antagonists suppress estrogen levels beyond those achieved alone [193]. Due to the interesting pharmacological profile of GnRHR antagonists, a number of non-peptidic (heterocyclic) molecules sharing various pharmacophores, such as, for example, substituted thieno[2,3-*b*]pyridine-4-ones, thieno[2,3-*d*]pyrimidin-2,4-diones, pyrrolo[1,2-*a*]pyrimidin-7-ones, imidazolo[1,2-*a*]pyrimidin-5-ones or uracil derivatives, have been synthesized [199,200,201].

The GnRHR agonist-binding domain seems to be defined by, amongst others, the Asn-102 (helix 2), the Lys-121 (helix 3) and the Glu-301 (helix 7). Whereas the Asn-102 interacts with the C-terminus amide function of the ligands, the Lys-121 interacts with the His-2 or Trp-3. The Glu-301 seems not only to interact with the Arg-8 through electrostatic contacts but also to modify the ligand conformation [190]. Lastly, it has been proposed distinct agonist and antagonist binding-sites, as the Lys-121 is not involved in the interaction of antagonists [202,203]. 

### 2.8. Somatostatin Receptors

**Figure 9:**
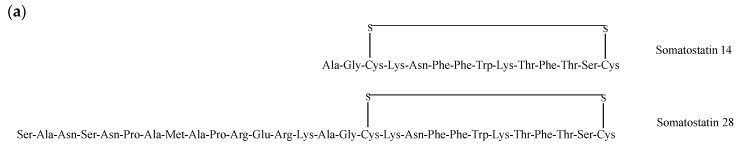

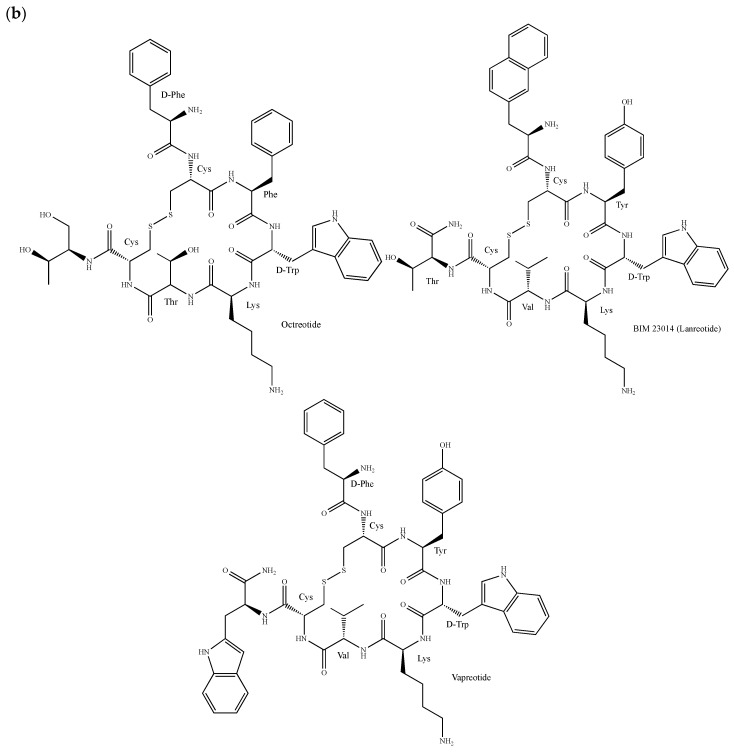
Structure of (**a**) somatostatin-14 and -28 and (**b**) the inhibitors octreotide, BIM 23014 and vapreotide.

Somatostatin (SST or SRIF for somatotropin release-inhibiting factor) is an inhibitory Trp-centered β-turn-containing cyclopeptide that exists under two forms, one of 14 amino acids (Somatostatin-14 or SRIF-14) and one of 28 amino acids (Somatostatin-28 or SRIF-28), as shown in Figure 9a. Produced in the hypothalamus, it functions not only as a neurotransmitter but also as an autocrine/paracrine hormone [204]. In addition, it exerts anti-proliferative effects on both normal and tumor cells [205]. The biological effects of SST are mediated by five receptor subtypes (SSTR1–5), which are expressed in a variety of tumors including breast cancer [206]. As such, they are attractive targets for the imaging and treatment of diverse tumors including neuroendocrine tumors (NETs) [207]. SST analogues, which show prolonged half-life, enhanced receptor subtypes selectivity and increased potency, control tumor growth and metastatization either directly by inducing cell cycle arrest, apoptosis and cell invasion suppression or indirectly by suppressing angiogenesis and the secretion of growth-promoting hormones and growth factors [208].

The synthetic SST analogue octreotide (Sandostatin^®^, Figure 9b) is highly effective in inhibiting hormone secretion and is used for the treatment of various endocrine and malignant disorders including NETs, acromegaly and gastroenteropancreatic tumors [209,210,211]. Moreover, it shows inhibitory activity against breast cancer growth in nude mice and in cultured cells either alone or in combination with other drugs [212,213]. This peptide is composed of eight amino-acids, including a non-proteinogenic 2-amino-1,3-dihydroxybutyl and the pharmacophore Phe–Trp–Lys–Thr. With the help of a d-Trp, it is cycled through a disulfide bond to mimic the SST turn conformation [214]. The proliferation of breast cancer cells is also inhibited by the octreotide derivatives BIM 23014 (lanreotide, Somatuline^®^, Figure 9b) and vapreotide (Sanvar^®^, Figure 9b), and the doxorubicin-containing molecules AN-162 and AN-238 [215,216,217,218]. Phase II studies in breast tumor patients have demonstrated that a continuous subcutaneous infusion of vapreotide, which is commonly used in the treatment of AIDS-related diarrhoea and esophageal variceal bleeding in cirrhotic liver disease patients, can reduce serum levels of growth factors such as prolactin and the insulin like growth factor I (IGF-I), thus encouraging studies combining vapreotide with anti-estrogens, cytotoxic or anti-angiogenic agents [219]. Gratifyingly, a meta-analysis study of phase I and II trials indicates that in patients with metastatic breast cancer, the treatment consisting of the use of somatostatin analogues as first-line therapy is associated with a tumor response of over 40% with few side effects [220]. A number of radiolabeled SST analogue conjugates have also been synthesized not only for treatment but also for diagnostic applications [221,222]. For instance, a ^111^In-/^90^Y-labeled SST analogue, namely DOTA-d-β-Nal^1^-lanreotide (Dotalan^®^), has been shown to bind to a broad range of primary human tumors and tumor cell lines including breast cancer cells, presumably through SSTR2-5 [223].

### 2.9. Gastrin-Releasing Peptide Receptors

**Figure 10:**
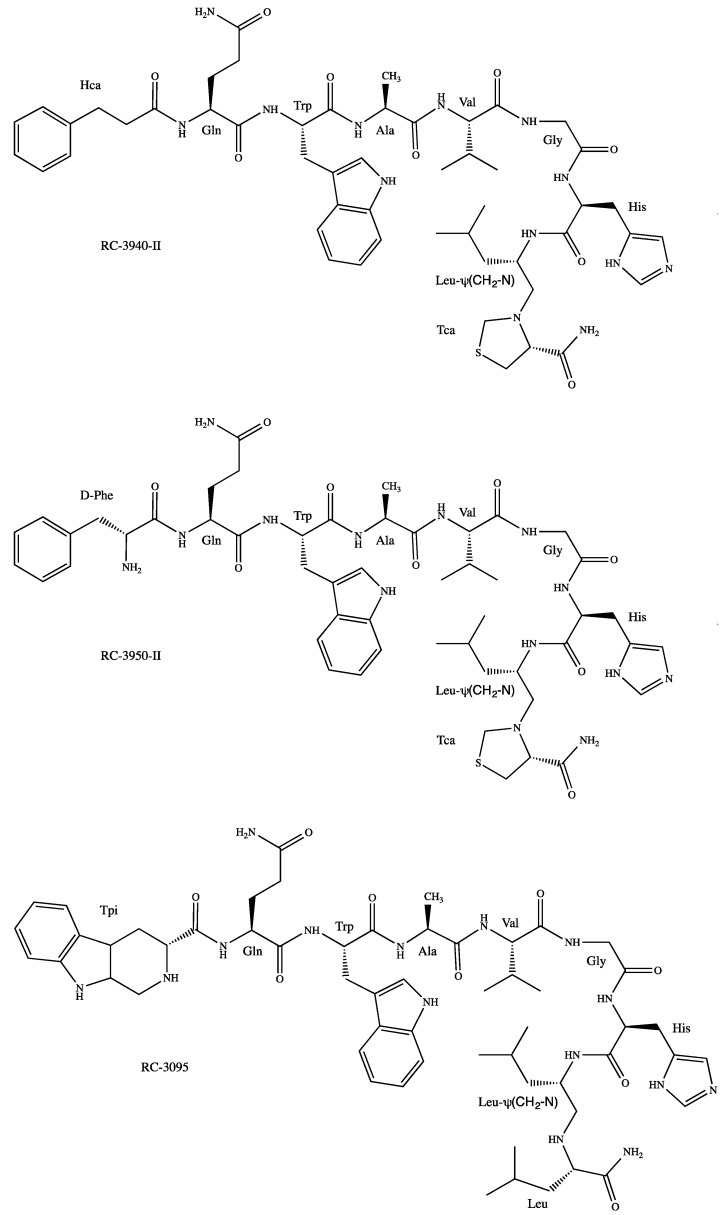
Structure of the GRP-R antagonists RC-3940-II, RC-3950-II and RC-3095, where Tpi corresponds to tetrahydro-1H-pyrido[3,4-*b*]indole, Hca corresponds to desaminophenylalanine and Tac corresponds to thiazolidine-4-carboxylic acid.

The mammalian gastrin-releasing peptide (GRP) is a neuropeptide of 27 amino acids that shares the same C-terminal sequence with the tetradecapeptide bombesin (i.e., His–Leu–Met–NH_2_). It interacts with a specific receptor, namely GRP-R, to exert several physiological actions including the release of peptide hormones, the secretion of gastric acid, the thermoregulation of enteric motor functions, circadian rhythm, smooth muscle contractions and immune functions [224]. In a high percentage of cancer tissues including breast tumors, GRP-R is overexpressed, allowing the use of GRP-like peptides labeled with β- and/or γ-emitting radionuclides for diagnostic purposes [225,226]. By using single-photon emission computed tomography (SPECT) and positron emission tomography (PET), several studies have demonstrated the usefulness of radiolabeled GRP-R ligands in mouse breast cancer models as well as in estrogen receptor (ER)-positive breast tumor patients [227,228,229,230]. Considering that GRP receptors are overexpressed in a high percentage of ER-positive breast tumors [231], GRP receptor imaging might be used successfully for disease staging and therapy evaluation in ER-positive breast tumor patients. Moreover, GRP elicits mitogenic effects in a number of cancerous tissues including breast tumors [232,233]. Even if there is a limited number of synthetic antagonists, the molecules RC-3940-II (Hca^6^,Leu^13^Ψ(CH_2_-N)Tac^14^-bombesin(6-14)], RC-3950-II [d-Phe^6^,Leu^13^Ψ(CH_2_-N)Tac^14^-bombesin(6-14)) and RC-3095 (Figure 10), which all encompass the bombesin fragment Asn–Gln–Trp–Ala–Val–Gly–His–Leu–Met (residues 6–14), have been shown to decrease the volume of breast cancer xenografts, thus opening new perspectives for the treatment of breast tumors [234,235,236].

### 2.10. Endothelin Receptors

Endothelins (ET-1, ET-2 and ET-3) are 21-mer peptides containing two stabilizing disulfide bonds. They elicit a number of biological responses by binding to two GPCRs known as endothelin receptor type A (ETAR) and type B (ETBR) [237]. Beyond being the most potent vasoconstrictor in the human cardiovascular system, ET-1 signaling elicits pleiotropic effects in cancer cells and the sourrounding microenvironment by affecting cell proliferation, apoptosis, migration, EMT, chemoresistance and neovascularization [237,238]. Furthermore, an elevated expression of ET-1 and ETAR in breast carcinoma patients is associated with lower survival [239], VEGF expression and angiogenesis [240] and predicts an unfavorable response to neoadjuvant chemotherapy in locally advanced tumors [241].

**Figure 11:**
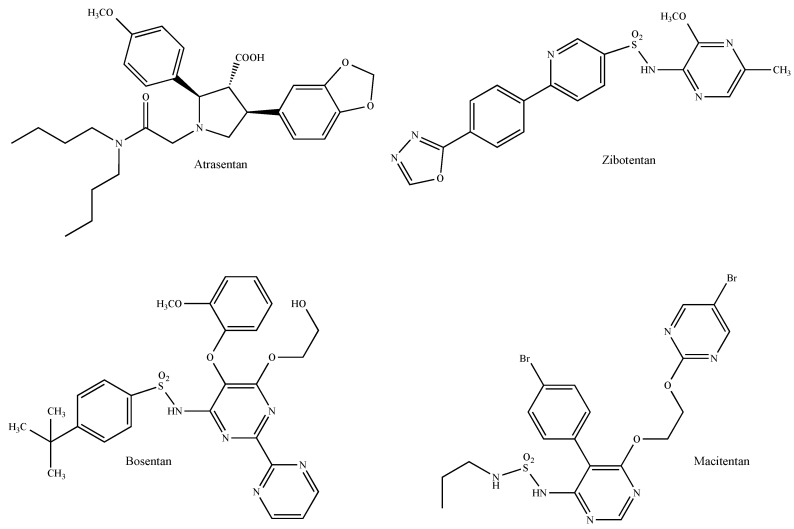
Structure of the ETAR antagonists atrasentan, ziboentran, bosentran and macitrentan.

The use of the selective ETAR antagonists atrasentan (ABT-627, Xinlay^®^ Figure 11) and zibotentan (ZD4054, Figure 11) as well as the dual endothelin receptor inhibitors bosentan (Tracleer^®^, Figure 11) and macitentan (ACT-064992, Opsumit^®^, Figure 11) represents the most promising approach for the control of the progression of ET-1-mediated tumors [242]. For instance, atrasentan reduces breast cancer cell invasiveness induced by hypoxia-mediated ET-1 secretion [243] and potentiates the anti-proliferative and anti-invasive effects of the HER2 antibody trastuzumab in HER2-overexpressing breast cancer cells [244]. In the same context, zibotentan exhibits additive anti-migratory and anti-invasive effects when concomitantly used with fulvestrant and aromatase inhibitors on breast cancer cells in vitro and in vivo [245]. The dual antagonist bosentan reduces tumor growth and decreases the levels of pro-inflammatory cytokines and pro-migratory endogenous molecules in immunocompetent mice implanted with mammary carcinoma cells [246]. Likewise, the bosentan derivative macitentan, which represents the next generation of orally active ET receptor antagonists with improved efficacy and tolerability [247], sensitizes experimental breast cancer brain metastases to paclitaxel [248]. Preclinical data obtained with dual ETAR and ETBR antagonists in diverse tumors suggest, therefore, that this class of drugs could be a promising therapeutic option for cancer treatments given that they can target not only tumor cells, which typically express ETAR, but also components of the cancer-associated microenvironment such as vascular, lymphatic and inflammatory cells and fibroblasts, which all express ETBR [238].

### 2.11. The Hedgehog and Wnt Signaling Pathways

**Figure 12:**
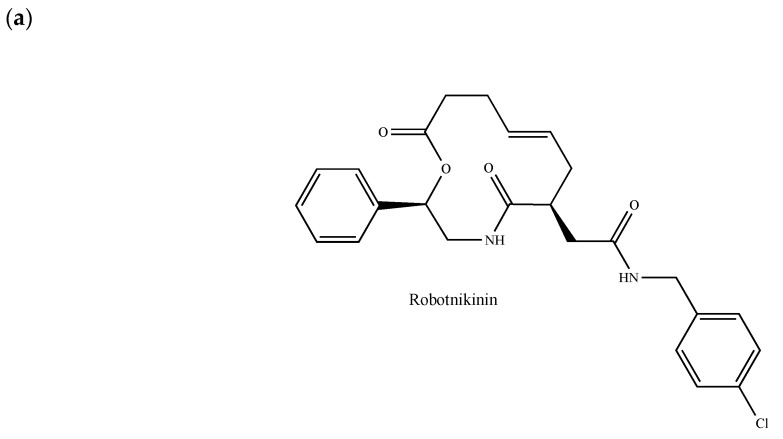

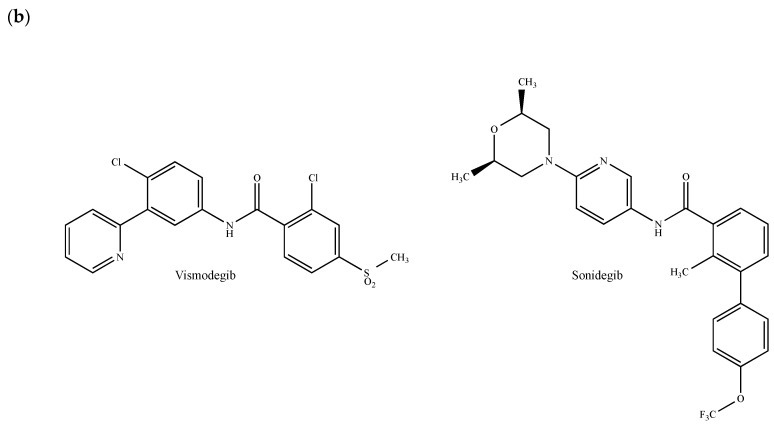
Structure of the compounds interfering with Hedgehog pathways: (**a**) robotnikinin, which binds the extracellular Sonic Hedgehog (SHH) protein and (**b**) vismodegib and sonidegib.

The Hedgehog (Hh) pathway plays important roles in embryonic patterning, stem cell renewal and tissue regeneration and repair [249]. It activates a signaling cascade mediated by the three secreted ligands called Sonic Hedgehog (SHH), Indian Hedgehog (IHH) and Desert Hedgehog (DHH), the 12-pass transmembrane receptor Patched1 (PTCH1) and the GPCR Smoothened (SMO). The glioma-associated oncogene (GLI) transcription factors, which are the effectors of Hh signaling, regulate the expression of target genes, some of which are involved in cancer cell proliferation survival, invasion, migration, epithelial-mesenchymal transition, angiogenesis, metastases and drug resistance [249,250]. More recently, it has been demonstrated that Hh signaling pathways are not only involved in the development of nornal mammary gland, but also in the growth of breast cancer [251]. In patients with invasive ductal breast carcinoma, Hh overexpression is associated with an increased risk of metastasis and with the emergence of a basal-like phenotype [252]. In a mouse model of basal breast cancer, Hh ligands increased tumor growth, induced a poorly differentiated phenotype, accelerated metastasis and reduced survival [252]. Given the contribution of Hh signaling-related processes in a multitude of cancers including breast cancer, several components of this pathway have attracted a great deal of interest as therapeutic targets [250]. Hh signaling pathways can be modulated at different levels: (a) by inhibiting ligand–receptor interactions through antibodies or robotnikinin (Figure 12a); (b) by directly interfering with the SMO; (c) by regulating ciliogenesis and ciliary localization of pathway components and (d) by targeting GLI transcription factors [249]. In this paragraph, we will exclusively pay attention to emerging small molecules antagonizing the protein SMO. Vismodegib (GDC-0449, Erivedge^®^, Figure 12b) and sonidegib (LDE225, Odomzo^®^, Figure 12b), which both received FDA approval for the treatment of basal cell carcinoma, inhibit breast cancer cell growth in vitro and in vivo [253,254,255]. In a phase I clinical trial (GEICAM/2012-12, EDALINE), three of 12 patients with metastatic TNBC were shown to benefit from the combination therapy of sonidegib and docetaxel, with one patient experiencing a complete response [256,257]. Further phase I trials demonstrated that sonidegib in combination with paclitaxel is well-tolerated and may exert antitumor effects in patients with advanced solid tumors including breast cancer [258]. Similar effects were observed in combination with docetaxel in advanced TNBC patients [256,259]. Of note, both vismodegib and sonidegib are currently under clinical investigation in patients with breast tumors and several other SMO antagonists are currently entered in clinical trials for the treatment of various cancer types.

Wnt proteins are a group of highly conserved secreted glycoproteins that are critical for embryo development and adult tissue homeostasis. Frizzled (FZD) proteins, which are seven transmembrane receptors for Wnt ligands and the Wnt co-receptors (namely low-density-lipoprotein receptor-related proteins 5/6 (LRP5/6)) are required for Wnt β-catenin-dependent and -independent signaling pathways [260,261]. The overactivation of Wnt signaling pathways is also implicated in several human diseases including colorectal, hematological and breast cancers [262]. Wnt signaling is activated in over 50% of breast cancer patients and is associated with reduced overall survival [263] and metastasis in TNBCs [264]. Moreover, canonical Wnt ligands and receptors, including FZD7, are frequently overexpressed in breast cancers, while secreted antagonists are silenced [265,266,267].

Several strategies targeting Wnt signaling in tumors, such as mimics of endogenous Wnt ligands and anti-FZD antibodies, are currently in the clinical testing stage [262,268]. For instance, the inhibition of porcupine (PORCN), which is a membrane-bound *O*-acyltransferase that is required for Wnt secretion and activity, has been indicated as an effective treatment for Wnt-dependent cancers. Accordingly, diverse PORCN inhibitors suppress the growth of mammary tumors in MMTV-Wnt1 transgenic mice [269,270]. Similar inhibitory effects were observed in MMTV-Wnt1 tumors treated with the soluble Wnt inhibitor Fzd8CRD, which consists of the fusion of the Fc region of IgG and the extracellular domain of the receptor FZD8, and LRP6 antagonists or anti-LRP6 antibodies [266,271]. In line with clinical observations revealing that the loss of expression of the protein Wnt-5a in primary breast tumors is associated with a faster tumor spread, the Wnt-5a-mimicking hexapeptide Foxy-5 (sequence: N–Formyl–Met–Asp–Gly–Cys–Glu–Leu–COOH) causes a significant reduction of breast tumor metastases in immunodeficient and immunocompetent mice [272]. Finally, a monoclonal antibody to FZD receptors (OMP-18R5, Vantictumab^®^) was shown to block the canonical Wnt pathway and to suppress breast cancer cell growth in a human tumor xenograft [273].

## 3. Conclusions

GPCRs play an important role in regulating breast cancer initiation and progression as they influence cell survival, growth, migration and metabolism. The overexpression of diverse GPCRs is associated with tumorigenesis and/or metastasis in diverse types of cancer tissues including breast tumors. As such, GPCRs represent attractive targets in breast cancer prevention and treatment. In this review, we have summarized the current knowledge on the action of certain GPCRs in breast cancer and discussed the benefits of GPCR-blockade strategies in this pathological context. Although GPCRs are the largest family of targets for approved drugs, these membrane proteins are of growing interest for the development of anticancer therapies and constitute an expanding field of medical research with exciting perspectives. The evidences reviewed here strongly support the concept that GPCRs can potentially serve as effective targets for breast cancer therapy. In this regard, it should be noted that GPCR-interferring drugs that are currently used for the treatment of others pathologies have shown beneficial effects against breast tumors. This is the case, for instance, of fingolimod (a functional S1P modulator used for the treatment of multiple sclerosis), the EP4 antagonist grapiprant (a veterinary drug used against osteoarthritis in dogs), the antiplatelet PAR1 antagonist varopaxar, anti-HIV-1 drugs and chemokine receptors inhibitors maraviroc and vicriviroc, in addition to the sartan family members valsartan, candesartan and losartan, which are AT1R and AT2R inhibitors recommended for use in heart failure patients. Ongoing efforts to wholly appreciate the biological activities elicited by GPCRs toward breast tumorigenesis will likely facilitate the development of new innovative pharmacological strategies for treating breast cancer patients.
